# “You’re losing your Ghanaianess”: understanding malaria decision-making among Africans visiting friends and relatives in the UK

**DOI:** 10.1186/1475-2875-13-287

**Published:** 2014-07-27

**Authors:** Penny E Neave, Ron H Behrens, Caroline OH Jones

**Affiliations:** 1 Department of Public Health, AUT University, Auckland, New Zealand; 2 Department of Clinical Research, Faculty of Infectious and Tropical Diseases, London School of Hygiene and Tropical Medicine, London, UK; 3 Department of Disease Control, Faculty of Infectious and Tropical Diseases, London School of Hygiene and Tropical Medicine, London, UK; 4 Kemri-Wellcome Trust Research Programme, Kilifi, Kenya; 5 Nuffield Department of Clinical Medicine, Centre for Tropical Medicine, University of Oxford, Oxford, UK

**Keywords:** Imported malaria, Africans visiting friends and relatives, Chemoprophylaxis, Malaria prevention, Malaria diagnosis, Malaria treatment, Migrant health

## Abstract

**Background:**

In the UK, the majority of imported malaria infections occur in the London area among UK residents of African origin who travel to Africa visiting friends and relatives (VFRs). Effective malaria prevention measures are available but there is little understanding of the factors that enhance and constrain their use among VFRs.

**Methods:**

Semi-structured interviews were undertaken with Africans resident in London who visited friends and relatives in Nigeria and Ghana (n = 20) and with African VFRs recently treated for malaria (n = 6). Data collection took place between December 2007 and February 2011. Information on migration patterns and travel of respondents was collected and the data were analysed using a framework analysis approach.

**Results:**

Knowledge of the link between mosquitoes and malaria was high. Factors influencing the use of mosquito avoidance methods included knowledge about the local environment, perceptions of the inevitability of contracting malaria, and a desire to fit with the norms of host families. Previous experience of bed nets, and the belief that more modern ways of preventing mosquito bites were available deterred people from using them. Chemoprophylaxis use was varied and influenced by: perceptions about continuing immunity to malaria; previous experiences of malaria illness; the cost of chemoprophylaxis; beliefs about the likely severity of malaria infections; the influence of friends in the UK; and, the way malaria is perceived and managed in Nigeria and Ghana. Malaria treatment was considered by many to be superior in Nigeria and Ghana than in the UK. A conceptual framework was developed to illustrate the manner in which these factors interact to affect malaria decisions.

**Conclusions:**

The use of malaria prevention among VFRs needs to be understood not only in terms of individual risk factors but also in relation to the context in which decisions are made. For VFRs, malaria decisions are undertaken across two distinct social and environmental contexts and within the structural constraints associated with each. Strategies for reducing the burden of malaria among VFRs that ignore this complexity are likely to face challenges. New approaches that take account of contextual as well as individual factors are required.

## Background

Most cases of imported malaria in high-income countries affect first or second generation migrants who have returned from ‘visiting friends and relatives’ (VFRs) in malaria-endemic countries [1]. In the UK, the majority of infections occur in the London area among UK residents of African origin who travel to Africa as VFRs [2]. London is home to large Nigerian and Ghanaian communities and over 50% of reported imported falciparum malaria infections were acquired in these two countries between 1987 and 2006 [2]. Effective malaria prevention measures such as chemoprophylaxis and the use of insecticide-treated mosquito nets (ITNs) are available and recommended by the UK’s Health Protection Agency for people travelling from the UK to malaria-endemic areas [3], but there is little understanding of the factors that enhance and constrain their use among VFRs. To date, most of the research on factors influencing the prevention and treatment of malaria among this group has focused on identifying and measuring individual risk factors such as perceptions of the risk of acquiring malaria and use of chemoprophylaxis [4]. However, little attention has been paid to the contexts within which decisions about malaria are made. One UK-based study which did explore the context of malaria chemoprophylaxis decision making found that VFRs were influenced by their previous experience of malaria episodes as well as by how malaria was managed in malaria-endemic countries [5]. A second US-based study reported that many migrants were critical of malaria services offered in the USA, when compared to those available in their country of birth [6]. These two studies suggest that the experiences of VFRs and the context in which they make decisions are important factors influencing their malaria prevention and treatment practices. Greater understanding of the experiences of VFRs and the context within which they make malaria-related decisions is essential to help identify new approaches to reducing the burden of malaria in this group.

To increase understanding of the context of malaria decision making among VFRs a study was undertaken to explore the perceptions and practices of VFR travellers and patients of Nigerian and Ghanaian origin living in London and of the health services available to them before travel and on their return. Data on the views of healthcare workers and the provision of malaria services in London have recently been published [7]. This paper explores the malaria perceptions and practices of the VFRs and describes how a range of individual and contextual factors influences their decision-making.

## Methods

### Study setting and participant selection

This qualitative study was undertaken in the London boroughs of Newham, Barking and Dagenham, Greenwich, Lewisham, Croydon, Merton, Lambeth, Southwark, Lewisham and Islington. Participants comprised VFRs and VFR patients (VFRPs) recently treated for falciparum malaria. Sampling was purposive with VFRs selected on the basis that: they were London residents; first or second generation Nigerian or Ghanaian migrants; and about to, or had recently visited friends and relatives in Nigeria or Ghana. Recruitment was through local African community groups, a local African health forum and by a request in a newsletter sent to employees in one Primary Care Trust. The VFRPs were subject to the same selection criteria as the VFRs and were recruited from two London hospitals. Patients with suspected falciparum malaria were encouraged to participate by the clinician treating them once all clinical care had been completed. For those who agreed, it was established that each was a laboratory-confirmed case of falciparum malaria.

### Data collection

Semi-structured interviews were conducted by the first author (PN) with three groups of participants: i) VFRs pre- and post-travel; ii) VFRs post-travel only; and iii) VFRPs on their discharge from hospital. Interviews were carried out at a time and location that was convenient for the participants. The VFRs interviewed pre-travel were requested to contact PN on their return, either by telephone or email, to provide additional information on their experiences during their trip. The VFRPs were contacted by telephone by PN after their discharge from hospital to arrange an interview.

Face to face interviews took between 30 and 50 minutes. Demographic data, migration and travel information were collected from each participant. The pre-travel interviews with VFRs and the interviews with the VFRPs were structured around a topic guide covering issues such as: malaria transmission, perceptions of risk, mosquito control methods, factors impacting on the uptake and adherence to chemoprophylaxis, malaria symptoms and management. Study participants were given the opportunity and encouraged by the interviewer to ‘tell their own story’ of their most recent and previous travel and experiences. Upon return, respondents were requested to confirm if they had carried out mosquito avoidance measures as planned, about their use and adherence to chemoprophylaxis and about malaria-related symptoms and treatment if these had occurred. Interviews were recorded using a digital voice recorder and were transcribed verbatim. Transcripts were exported into NVivo version 7. A framework analysis was undertaken and a conceptual framework was constructed.

Ethical approval to carry out the study was received from the London School of Hygiene and Tropical Medicine’s Ethics Committee (reference 5086).

## Results

Twenty-six participants were recruited and interviewed between December 2007 and February 2011. These comprised: 11 VFRs who were interviewed between one and four weeks before travel and between one and four weeks after travel; three VFRs who were interviewed before travel but who did not respond to post-travel email or telephone contact; six VFRs who were interviewed after travel only; and six VFRPs who were interviewed between one and three weeks after discharge from hospital.

Two participants who did not fit the original selection criteria were also interviewed. One was a VFR of French/Mauritanian origin who made frequent visits to Nigeria to visit friends and family and the other was a VFRP of Sierra Leonean origin who had recently visited relatives in Nigeria.

Demographic and travel-related information for VFRs and patients are shown in Additional files 1 and 2. These demonstrate that there was considerable heterogeneity within this small sample.

The median duration of travel by VFRs was 23 days (interquartile range 14–30 days), and for VFRPs it was 17 days (interquartile range 14–21 days). Five VFRs worked in healthcare, and three of these had some professionally acquired knowledge of malaria, while a sixth VFR was medically qualified. No VFRP had any professional knowledge of malaria. The majority of VFRs (18/20) had planned their last trip at least eight weeks in advance. By contrast, only one VFRP had planned their trip this far in advance. One decided to travel two weeks before the start of the trip while the remaining four all travelled within one week of deciding to do so.

### Malaria risk and mosquito avoidance

All participants understood the potential risk of acquiring malaria in Nigeria and Ghana and were aware of the transmission route. Many explained that they always stayed with the same friends or relatives, or in their own homes, and these locations were not close to mosquito breeding sites. Despite this assurance, a few participants acknowledged that it was sometimes difficult to rely on neighbours to maintain adequate sanitation, which limited the effectiveness of their own efforts. Some of the older respondents were surprised by the current poor sanitation they witnessed, and recalled memories of regular community-organized cleaning of mosquito breeding sites.

All but one VFR interviewed before travel described the spraying of bedrooms with insect repellents as a regular practice carried out by their host family, which took place between 30 to 60 minutes before bed time. However, nine of the 11 contacted upon return to London reported that spraying had not been carried out routinely. They were not concerned however, as they believed that the window netting was adequate, and they had not been troubled by mosquitoes.

It was widely recognized that the measures used to avoid mosquitoes were not always effective, particularly if time was spent sitting outside in the evening (sometimes to avoid the smell of the insect repellent), if there were power cuts, or if short visits were made to more rural areas where housing was of a lower standard and electricity rare. One measure that several of the VFRs reported using to prevent being bitten by mosquitoes, which they said were not used by their hosts, was body creams containing insect repellent. The use of these repellents was most commonly reported by those travelling with children.

Questions about the use of bed nets were met by the majority of VFRs and VFRPs with amusement. Few considered them an acceptable form of prevention against mosquitoes. Of the seven VFRs travelling with children, only three planned to provide bed nets for them. Two others would consider their use; one, only if visiting a rural area; the other in places where window nets were not available. Upon return to the UK, of those three who intended to use nets, one reported that in fact her children did not do this, and she had indicated their unwillingness to do so in the pre-travel interview. Another was lost-to-follow-up and the third considered it to be too much effort to put up the net, particularly as air conditioning was available as an alternative means of avoiding mosquito bites.

The most commonly mentioned reason for not using a net was connected to unpleasant childhood memories of sleeping under one. Participants explained that they exacerbated the heat and closeness of an already uncomfortable atmosphere. Their use was also discussed as a practice associated with times long since passed, now replaced with modern, more effective (and pleasant) methods, for example air conditioning and extensive netting around doors and windows. In contemporary Nigeria and Ghana they were described as only being used for children.

### Chemoprophylaxis

There was considerable variation among the respondents in their reported use of chemoprophylaxis (Additional files 1 and 2). Although some regularly used it, some did not, and some who would use it on this trip had not done so previously. None of the six VFR patients reported having used chemoprophylaxis even though four of these six lived in a borough where chemoprophylaxis purchase was subsidized, and one was unaware that subsidized chemoprophylaxis was available.

### Experiences and perceptions of chemoprophylaxis

With no prompting from the interviewer, the cost of chemoprophylaxis was often raised as an issue by many participants. Many were of the opinion that they and their friends and family living in the UK considered the cost to be prohibitive or not worth the money, especially compared to the cost of malaria treatment in Nigeria or Ghana. Of the eight VFRs who reported that they always used chemoprophylaxis, four (three of whom lived in areas of London where the drugs were not subsidized) said that the price was personally difficult to afford. One VFR (VFR11) considered the cost of mefloquine to be just about justifiable, but would not purchase the more expensive atovaquone-proguanil (AP), particularly given her other concerns:

“*so the person said I would have to pay, I think £3 something per tablet and it would come to about £150 or something like that. So I said there’s no way I’m paying that much for anti-malarial pills, so I went to get it changed and then got the weekly ones, which was fine because I don’t like taking medicine that often anyway.*” (VFR11)

Other VFRs mentioned that the cost of chemoprophylaxis was not an issue for them if and when they were travelling alone, but that when travelling with other family members the cost could become prohibitively expensive.

Access to chemoprophylaxis was reported to have been a barrier to its use among two of the six VFRPs. Both reported that they tried to purchase AP through local pharmacies but because they did not have a prescription, they were unsuccessful.

A further barrier to the use of drugs as chemoprophylaxis described by several of the VFRs was previous experiences of unpleasant side effects when using chloroquine. Several participants remembered that they had used (or been given) this drug to treat infections they had suffered during childhood. The intense itching they experienced while using this treatment appeared for many to be a sufficient disincentive to take chemoprophylaxis due to concerns that these drugs might contain chloroquine or other substances that had the same effects.

A final issue that arose either directly or indirectly in relation to malaria avoidance behaviours in general, and the use of chemoprophylaxis in particular, was the feeling that using these protective measures was an over-reaction to a ‘normal’ illness. Overt concern about malaria marked them out as different to the local population in the country that they were visiting. This was expressed in several ways. For one participant her newly acquired caution about malaria and its avoidance had caused an argument with her mother (who lived in Ghana):

‘…*but when we from here* [the UK] *go there* [Ghana] *they say that we’re doing things extreme, you know, we’re being so protective and all that yeah, but them, it’s a normal thing to them because….’*

Do you find it awkward when they think you are over the top?

‘I do, I always argue with my mum’

So she says you don’t need to bother with it all?

*‘Sometimes.* [her mother says] *You were born here so stop being like that, you know.’* (VFR7)

A second suggested that their concerns might cause problems and disruption to their hosts:

‘.. *if you go to a village situation they think you’re over-reacting. They say it’s a lot of fuss, what’s it all about? Because you are just putting a lot of problems on their finances, you know.’* (VFR1)

Another mentioned that overtly expressing concern about malaria or using chemoprophylaxis while visiting Nigeria was likely to result in being laughed at, although not directly:

Would you think your friends and relatives in Nigeria would be surprised if you…. started taking chemoprophylaxis?

‘Yeah, I think some people would. I mean my parents probably wouldn’t, obviously they want the best for me, but there would be a bit of sniggering in the background..’

Not from your parents, or maybe from your parents?

‘Maybe from them, but in the bedroom away from me. Everyone would probably snigger’

Why do you think they would snigger?

*‘Oh just because they think you know we live here we’re not dead from this stuff so what makes you think just because you’ve lived away from us for a few years you’ve suddenly become so susceptible that and even if you did have malaria what’s the worst that can happen other than a few days in bed?”’* (VFR 16)

While a fourth suggested that in using chemoprophylaxis she was demonstrating her difference and perhaps moving away from her origins, or shedding something of her African identity:

What about friends in Ghana, would they think you were weird if you took chemoprophylaxis drugs?

*‘Not weird, but yeah it sets you apart from the rest, ‘cos* [they say] *you’re still Ghanaian why do you need to?* ***……’***

Would it make you feel less Ghanaian, if you started taking anti-malarials and doing all these things?

‘It doesn’t make me, but I can see how that would..’

Do you think it’s an age thing as well?

‘No. ‘Cos my dad makes fun of me as well.’

So what does he say?

‘You’re losing your Ghanaianess. He always says that.’

So it’s just something to tease his daughter about*?*

*‘Exactly.’* (VFR12)

### Perceptions of susceptibility and consequences of infection

There were considerable differences among the participants in their perceptions of their personal susceptibility to malaria. For example, two of the VFRs, based on memories that despite sharing the same sleeping space they had contracted malaria as children although their siblings had not, suggested that they were particularly susceptible to the disease. A third believed he was susceptible to being bitten because he had “sensitive skin”. Another however described malaria as “not being my disease”, whilst two young male VFRs referred to their current good health and physical strength to explain how they would be able to tackle any malaria infection, albeit with the use of drugs.

A gradual loss of acquired immunity to malaria after leaving a malarious country was recognized by some VFRs and VFRPs. Experiences of more recent episodes of malaria (either personal or in other VFRs), were often described as being more serious than those acquired whilst living in Nigeria or Ghana and had begun to make some respondents consider using chemoprophylaxis for future visits. On the other hand, others perceived that, despite having not lived in a malaria-endemic region for several years, they retained at least some immunity, especially if they travelled regularly:

In terms of the health concerns that you got, you mentioned, you know, food, water, insect bites, are there any of those that would be a priority or are they pretty much similar in terms of priority?

*‘I don't know, because I travel regularly now, so I always feel I’m one of them. So I mean, people talk about umm defences, about, what's the word I'm trying to look for? People talk about the defences of the body, so I, when you go regularly you’re part of it. Perhaps, when I first started going, about once every four years, I used to take a lot of concern with umm, you know insect bites, you know, making sure I've got my malaria tablets, or doxycycline, but these days because I go regularly now, so it's like I've got my, I’ve always, you know, been used, I've built up umm, built up my own defence in my body.’* (VFR8)

All but two participants reported that they had previously experienced illnesses that they described as being malaria whilst living in Nigeria or Ghana. Few recalled if these episodes were laboratory-confirmed and most described the illness as having been mild. A fatal outcome was considered theoretically possible but very unlikely and thought possible only if related to other factors or the result of not accessing treatment promptly. The importance of being aware that symptoms experienced could be due to malaria and swift treatment were consistently highlighted as key to a successful outcome. This was a trusted and common practice by participants, either when they lived in Africa, or travelled as VFRs. VFRs were asked what action they would take if they developed symptoms which they thought might be malaria when they were in Nigeria or Ghana. The plan most commonly described was that if symptoms were similar to those previously experienced, they would monitor their own condition for 24–48 hours. Twelve of the 20 respondents said that, following the initial monitoring period, if they were still sick, they planned to treat themselves. Of these, 11 would purchase drugs for self-treatment from a community pharmacist, and one would double her dose of the antibiotic doxycycline, the chemoprophylactic drug she always took. If symptoms persisted after self-treatment was tried, they would seek medical advice. The eight others would not treat themselves, but would seek medical advice if they had not recovered within 24–48 hours.

Among the VFRPs interviewed, symptoms of uncomplicated malaria were initially attributed by one as being caused by influenza, and another by stress or post-travel fatigue. The others speculated that they might have contracted malaria. All initially tried some form of symptom relief, including paracetamol, aspirin and ibuprofen. Two also took (SP) as advised by friends, before seeking medical advice. One patient also drank two bottles of Schweppes™ tonic water on the understanding that it contained quinine and had been used previously by his wife’s family in Ghana as a malaria treatment.

Although a few respondents stated the advantages of malaria management in the UK, with an emphasis on a laboratory-confirmed diagnosis, it was clear that many participants had significantly more faith in the way that malaria is managed in Ghana or Nigeria than in the UK. Buying substandard drugs from community pharmacists in Nigeria and Ghana was recognized as a risk, but it was felt this could be overcome by using their own or friends and relatives’ knowledge about trusted pharmacies, or purchasing treatment from a different pharmacy if symptoms persisted. Few were aware of the resistance of malaria parasites to previously effective drugs such as chloroquine and SP, and several VFRs mentioned SP as a cheap malaria treatment readily available in Ghana and Nigeria.

Several VFRs voiced criticisms of malaria care in the UK, based on either personal experience or witnessing hospital care for malaria in the UK for friends and relatives. Concerns cited were delays in diagnosis by doctors unfamiliar with the disease, the need for transfer to other hospitals where clinicians had more expertise and incorrect treatment. According to two respondents, several deaths would result if malaria treatment was managed in Nigeria and Ghana as it is in the UK. Concerns about being placed in isolation in hospital were also voiced by many. This was a policy believed to have been implemented because non-African clinicians did not understand that malaria was not directly transmissible. The reaction was incredulity that this easily managed illness in Nigeria or Ghana is dealt with in the UK in this unnecessary way. One VFR’s family had delayed her presentation at hospital in London to avoid this, whilst another described how he was encouraged by a friend to treat himself to avoid being isolated upon admission to hospital in the UK:

Yeah, so how do your friends and relatives over here feel about that?

‘Uh, they think it’s a joke. Because when I phone my friend in that, in the flight, my friend is telling me to come down the aircraft and get treatment over there before I fly back. I said I’d rather get back here and treat myself. He said but they’re gonna quarantine you for about a week, I said, I don’t care, as long as I get well, yeah.’

So it’s commonly accepted that people think you’re gonna get treated like that?

‘Yeah’.

So do your friends normally take tablets before they travel? [back to the UK]

*‘A lot of them do*.”’ (VFRP 6)

## Discussion

Among individuals of West African origin who are now resident in the UK but travel back to their countries of origin to visit friends and relatives, the risk of contracting malaria is a significant and ongoing health problem [2,8,9] and the reported use of measures to prevent malaria infection (eg, the use of chemoprophylaxis) in this group is low [2].

Most previous studies of the use of malaria preventive measures among VFRs have investigated individual level factors as the main driver of preventive behaviours [4]. This type of approach to understanding health behaviours has been widely criticized for tending to ignore the structural factors which constrain individual actions [10] and for not taking into account the full range of contextual factors that affect decision making about health [11-15]. The aim in this current study was to try and understand the malaria experiences and practices of VFRs from their own perspective and to situate their actions in the broader contexts within which they occur. Using the data gathered, the goal was to develop a conceptual framework demonstrating how a range of contextual and individual factors might constrain or enhance the uptake of effective malaria preventive and treatment measures among this group.

The sample size in this qualitative study was small and the participants were heterogeneous in terms of their place of birth, country of origin (although all were from West Africa), length of residency in and frequency of travel, but there appeared to be little systematic variation in their experiences and perceptions of malaria. All participants were aware that malaria is transmitted by mosquitoes and that they were potentially at risk from the disease when they visited Nigeria or Ghana. However, the majority of participants were of the view that the locations in which they would stay were themselves low risk in terms of malaria acquisition (staying in well screened houses in areas where there were few mosquitoes), although several did express concerns about being able to avoid all mosquito bites. The reasons given by respondents to explain why they did not like using nets were in common with those found in studies investigating reasons for the low use of nets in Nigeria and Ghana and include discomfort and perceptions that better methods to prevent mosquito bites are available [16-18].

All of the participants perceived that, even if they became infected, the disease would be relatively easily dealt with, particularly if the episode occurred while in Nigeria or Ghana. These perceptions appear to be influenced by the relationship between the traveller and location which they would be visiting; the participants were visiting friends and/or relatives and had trust in the fact that they would be in a safe, relatively familiar environment, with people around to help them should they become ill. This feeling of ‘security’ , particularly in relation to malaria risk, was reinforced by their previous experiences with the disease and the common perceptions and experiences of malaria among the friends and relatives whom they visited in Nigeria and Ghana, that malaria is a mild illness that is easily and cheaply treated [19-21] with most cases being diagnosed and treated by pharmacists or by self-diagnosis on the basis of clinical symptoms, without recourse to parasitological confirmation of malaria [19]. Drugs for treatment are commonly purchased from retail outlets [20,22]. Few participants discussed the limitations of this approach to malaria diagnosis, that is, that malaria cannot be reliably diagnosed from clinical symptoms alone, and may result in misdiagnoses and delayed treatment, and that this can be fatal for those infected with *Plasmodium falciparum*.

The participants in this study all strongly contrasted the perceptions and approach to malaria that they had experienced in Ghana and Nigeria to those that they had experienced in the UK. In the UK, malaria is depicted as being a ‘medical emergency’ [23], a killer disease which needs to be avoided at all costs and, once a person is infected they must receive specialized medical treatment that can only be accessed through a series of highly regulated medical professionals. These contrasting approaches illustrate two of the key limitations of addressing malaria in VFRs as a purely individual biomedical problem without consideration of contextual and structural factors. Firstly, the data demonstrate that while the disease malaria is a medical condition caused by a naturally occurring parasite eliciting a variety of biological responses within individuals, the way in which malaria is experienced and managed varies according to context. That is, the approaches to malaria prevention and responses to an episode of malaria illness are socially constructed [24,25]. Secondly, the experiences of VFRs suggest that the structure of the health system itself shapes responses to illness episodes.

**Figure 1:**
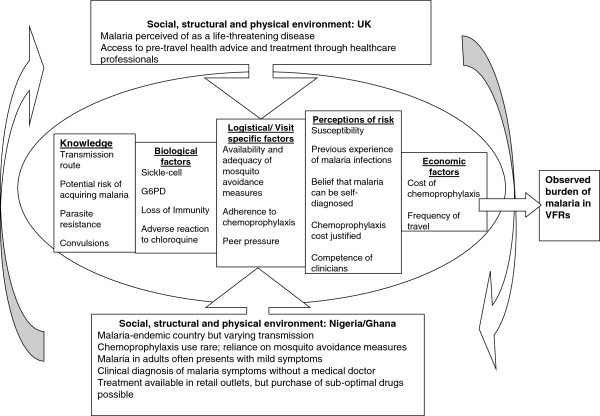
Conceptual framework illustrating contextual and individual factors that constrain or enhance the uptake of effective malaria preventive and treatment measures among Nigerian and Ghanaian VFRs.

The contrasting health systems and approaches to the disease and its management that are found in West Africa and the UK could be conceptualized as being at either end of a spectrum with the VFRs moving between the poles as they travel from one location to the other. In addition to the evidence that the VFRs move between these very different contexts when they travel, the data from this study agree with the findings from previous research which suggest that there are a large number of individual factors that contribute to malaria-related decision making among VFRs [4]. The conceptual framework (Figure 1) illustrates the range of factors within each location, the differences between location and the variety of individual factors that combine to shape malaria practices as individual VFRs move from the UK to West Africa and back again.

No one factor is likely to play a dominant role in decision making for all individuals at all times; rather different factors may have different prominence for the same individual at any given point in time depending on the context in which he or she finds themselves. In such complex situations, where a range of individual and contextual factors influence decision-making, single interventions that focus on one particular aspect of the decision-making process are unlikely to have a significant or consistent impact. For example, recent research has shown that subsidizing the cost of malaria chemoprophylaxis may have a slight impact on reducing the burden of imported malaria, but also suggested that no one single intervention is likely to significantly reduce the incidence [26].

While this research has pointed to the complex nature of malaria decision-making among VFRs, further exploration of the relationship between these individual and contextual factors and their influence on decision-making is essential to inform the development of intervention strategies that are targeted at the specific needs of VFRs as they circulate between the different landscapes of West Africa and the UK.

Furthermore, the results presented here raise fundamental questions about the control of infectious diseases such as imported malaria in early 21^st^ Century dominated by increased migration and travel. On the one hand, Angell and Cetron point out that the means exist in high-income countries with a low burden of infectious disease to prevent most travel-related illnesses such as malaria, but structural barriers, such as poor access to pre-travel health advice and prevention tools may be the barrier to achieving this [27]. On the other hand, as this research and that of others [26] has suggested, it may be that reducing structural barriers alone is not sufficient to achieve the control of diseases. As Gushulak points out, “many of the health threats, risks, and challenges related to health outcomes due to migration result from factors and influences present outside the jurisdiction and hence the direct influence, of the migrant-receiving nations” [28]. The findings in this study suggest that the malaria burden observed among VFRs in the UK is related both to structural factors within the UK and influences outside the jurisdiction of the UK. This suggests that public health practitioners need to be aware not only of the structural constraints within their own systems but also of the broader global context in order to develop different ways of approaching the problem of disease control in the 21^st^ Century.

## Conclusion

Previous research has identified a range of individual factors that impact on the burden of malaria among VFRs in the UK. This study, undertaken among VFRs living in London, suggests that their particular circumstances, straddling the different social, cultural and environmental contexts found in West Africa and the UK are central to their experiences and perceptions of malaria. The impact of these differences in context and the structural constraints present in each location are important factors affecting individual decisions about malaria prevention and treatment. Consideration of the differences that occur across the two domains and through time is essential to the development of effective strategies for malaria prevention among VFRs. Further research should be undertaken to determine if the findings from this study are relevant for other travel-related diseases which disproportionately affect African migrants.

This is one of few studies that have attempted to understand malaria decision making from the perspective of the VFRs themselves and the conceptual framework developed is a first attempt at distinguishing the key issues to consider in planning future research and developing appropriate strategies for the reduction of the burden of malaria among VFRs.

## Abbreviations

SP: Sulphadoxine pyrimethamine; VFR: Visiting friends and relatives; VFRP: Visiting friends and relatives patient; WHO: World Health Organization.

## Competing interests

RHB is a member of the advisory board of Sigma Tau and has received research funding from Sigma Tau. The authors have declared that there are no other competing interests

## Authors’ contributions

PEN jointly conceived the study, carried out the fieldwork, analysed the data and co-wrote the paper. COHJ jointly conceived the study, provided advice on the study design and analysis, and contributed to writing the paper. RHB jointly conceived the study, provided expert clinical advice and contributed to writing the paper. All authors read and approved the final manuscript.

## Supplementary Material

Additional file 1 VFRs demographic, migration and travel details, access to and use of chemoprophylaxis. Click here for file

Additional file 2 VFRPs demographic, migration and travel details, access to and use of chemoprophylaxis. Click here for file
