# Supplementary material for: “You’re losing your Ghanaianess”: understanding malaria decision-making among Africans visiting friends and relatives in the UK
Source: Malar J. 2014 Jul 27;13:287. doi: 10.1186/1475-2875-13-287 (PMC4118190; doi:10.1186/1475-2875-13-287)
Supplement: Additional file 1 — VFRs demographic, migration and travel details, access to and use of chemoprophylaxis. [file 1475-2875-13-287-S1.docx]

**Additional file 1: VFRs demographic, migration and travel details, access to and use of chemoprophylaxis**

| **VFR** | **Age range** | **Sex** | **Country of birth** | **Number of years resident in UK** | **Current frequency of travel to Nigeria/Ghana** | **Country visited on most recent trip** | **Access to subsidised chemoprophylaxis through area of residence** | **Use of chemoprophylaxis** |
| --- | --- | --- | --- | --- | --- | --- | --- | --- |
| 1 | 40-50 | M | France | 10 | Once or twice a year | Nigeria;  Ghana | No (and not registered with a GP) | No |
| 2 | 50-60 | M | Ghana | 22 | Once every year | Ghana | Yes | Intended to, but lost to follow-up |
| 3 | 30-40 | M | UK | 14 | Once previously | Ghana | No | No (intended to purchase it, but did not on his most recent trip). Had used it on one previous trip to a malarious country |
| 4 | 40-50 | M | Ghana | 22 | Twice. First time six years ago and once since then. | Ghana | Yes | Yes, intermittent previously |
| 5 | 50-60 | F | Ghana | 30 | Every two years | Ghana | No | Yes |
| 6 | 30-40 | M | UK | 4 | Every year | Nigeria | No | No |
| 7 | 40-50 | F | Ghana | 19 | Every two years | Ghana | Yes | Yes |
| 8 | 50-60 | M | UK | 25 | Once or twice a year | Nigeria | No | Provided contradictory information, and lost to follow-up |
| 9 | 40-50 | M | Nigeria | 2 | Four years since last visit, but previously once or twice a year (for business) | Nigeria | No | Yes |
| 10 | 20-30 | M | Nigeria | 8 months | 1^st^ time visiting since coming to UK | Nigeria | No | No |
| 11 | 60-70 | F | Nigeria | 35 | Every year | Nigeria | No | Yes |
| 12 | 20-30 | F | Ghana | 9 | Every year | Ghana | Yes | Yes, but intermittent previously |
| 13 | 30-40 | M | Nigeria | 30 | Twice or three times a year | Nigeria | Yes | Intermittent previously and lost to follow-up |
| 14 | 40-50 | F | Ghana | 19 | Every year | Ghana | No | Yes |
| 15 | 40-50 | F | UK | Since birth | Visited twice with gaps of 10 years between | Ghana | Yes | Yes |
| 16 | 30-40 | M | UK | 16 | Initially "infrequent travel", now "virtually every year" | Nigeria | No | Yes on this trip but intermittent on previous trips |
| 17 | 40-50 | F | Ghana | 20 | Once every two years | Ghana | No | Yes |
| 18 | 40-50 | F | Nigeria | 16 | Once every year | Nigeria | Unclear (two boroughs of residence given, one in an area offering subisidised chemoprophylaxis, one not | No |
| 19 | 30-40 | F | Ghana | 13 | Once every two years | Ghana | No | Yes |
| 20 | 20-30 | F | Nigeria | 1 ½ | 1^st^ time visiting since coming to UK | Nigeria | Yes | No |
