# Supplementary material for: “You’re losing your Ghanaianess”: understanding malaria decision-making among Africans visiting friends and relatives in the UK
Source: Malar J. 2014 Jul 27;13:287. doi: 10.1186/1475-2875-13-287 (PMC4118190; doi:10.1186/1475-2875-13-287)
Supplement: Additional file 2 — VFRPs demographic, migration and travel details, access to and use of chemoprophylaxis. [file 1475-2875-13-287-S2.docx]

Additional file 2: VFRPs demographic, migration and travel details, access to and use of chemoprophylaxis

| **VFRP** | **Age**  **Range** | **Sex** | **Country of birth** | **No of years resident in UK** | **Current frequency of travel to Nigeria/Ghana** | **Country visited on most recent trip** | **Access to subsidised chemoprophylaxis through area of residence** | **Use of chemoprophylaxis** |
| --- | --- | --- | --- | --- | --- | --- | --- | --- |
| 1 | 40-50 | M | Sierra Leone | 22 | First time since 2003 | Nigeria | Yes | No |
| 2 | 60-70 | M | Ghana | 24 | Once every two years | Ghana | Yes | No |
| 3 | 50-60 | M | Nigeria | 32 | Once or twice a year | Nigeria | Yes | No |
| 4 | 40-50 | M | Nigeria | 20 | Last year six times and “frequently” before then since finishing studies | Nigeria | Yes | Intended to use his usual protection (chloroquine and Proguanil purchased in the UK and kept in Nigeria), but no supplies remaining upon his arrival |
| 5 | 40-50 | M | Nigeria |  | First time since 2002 | Nigeria | No | No |
| 6 | 40-50 | M | Nigeria | 26 | Several times a year currently, upon arrival to UK yearly visits then gaps of three to four years | Nigeria | No | Intermittent, not on current trip |
